# Natural selection maintains species despite frequent hybridization in the desert shrub *Encelia*

**DOI:** 10.1073/pnas.2001337117

**Published:** 2020-12-14

**Authors:** Christopher T. DiVittorio, Sonal Singhal, Adam B. Roddy, Felipe Zapata, David D. Ackerly, Bruce G. Baldwin, Craig R. Brodersen, Alberto Búrquez, Paul V. A. Fine, Mayra Padilla Flores, Elizabeth Solis, Jaime Morales-Villavicencio, David Morales-Arce, Donald W. Kyhos

**Affiliations:** ^a^Department of Integrative Biology, University of California, Berkeley, CA 94720;; ^b^TruBreed Technologies, Oakland, CA 94609;; ^c^Department of Biology, California State University - Dominguez Hills, Carson, CA 90747;; ^d^School of the Environment, Yale University, New Haven, CT 06511;; ^e^Institute of Environment, Department of Biological Sciences, Florida International University, Miami, FL 33199;; ^f^Department of Ecology and Evolutionary Biology, University of California, Los Angeles, CA 90095;; ^g^Jepson Herbarium, University of California, Berkeley, CA 94720;; ^h^Department of Environmental Science, Policy, and Management, University of California, Berkeley, CA 94720;; ^i^Instituto de Ecología, Universidad Autónoma de México, Sonora, 83000 Hermosillo, México;; ^j^Reserva de la Biosfera el Vizcaíno, Guerrero Negro, 23941 Baja California Sur, México;; ^k^Benito Juárez s/n, Colonia Barrio La Punta, Bahia Asunción, 23960 Baja California Sur, México;; ^l^Department of Plant Biology, University of California, Davis, CA 95616

**Keywords:** adaptation, gene flow, hybrid zone, reciprocal transplant, speciation

## Abstract

Natural selection is an important driver of genetic and phenotypic differentiation between species. For species in which potential gene flow is high but realized gene flow is low, adaptation via natural selection may be a particularly important force maintaining species. For a recent radiation of New World desert shrubs (*Encelia*: Asteraceae), we use fine-scale geographic sampling and population genomics to determine patterns of gene flow across two hybrid zones formed between two independent pairs of species with parapatric distributions. After finding evidence for extremely strong selection at both hybrid zones, we use a combination of field experiments, high-resolution imaging, and physiological measurements to determine the ecological basis for selection at one of the hybrid zones. Our results identify multiple ecological mechanisms of selection (drought, salinity, herbivory, and burial) that together are sufficient to maintain species boundaries despite high rates of hybridization. Given that multiple pairs of *Encelia* species hybridize at ecologically divergent parapatric boundaries, such mechanisms may maintain species boundaries throughout *Encelia*.

## Significance

In Baja California, the deserts meet the coastal dunes in a narrow transition visible even from satellite images. We study two species pairs of desert shrubs (*Encelia*) that occur across this transition. Although these species can interbreed, they remain distinct. Using a combination of genetics, field experiments, three-dimensional imaging, and physiological measurements, we show that natural selection helps counteract homogenizing effects of gene exchange. Different habitats of these species create multiple mechanisms of selection—drought, salinity, herbivory, and burial, which together maintain these species in their native habitats and their hybrids in intermediate habitats. This study illustrates how environmental factors influence traits and fitness and how these in turn can maintain species, highlighting the importance of natural selection in speciation.

A major goal in biology is to understand the balance between natural selection and gene flow in the origin and maintenance of species. While natural selection is believed to play a role in the formation and maintenance of most species, under what conditions selection can generate and maintain phenotypic and genetic differences in the presence of high rates of gene flow is unknown (1–3). Under ecological speciation, adaptation to different habitats and trade-offs in resource use and allocation can generate strong divergent natural selection, manifested as low fitness of migrant and hybrid phenotypes (1, 4). Despite the appeal of adaptation as a robust mechanism of species formation and maintenance, population genetic models of divergence with gene flow require levels of natural selection that may be unrealistically high or uncommon in nature (5–7). To accommodate this discrepancy, many speciation models invoke the evolution of prezygotic barriers—either as a by-product of local adaptation (8) or independent of it—that “complete” speciation by locking in locally adaptive traits (7, 9). Particularly in sessile species like plants, local adaptation to divergent habitats can reduce the frequency with which different species encounter each other, thus further reducing gene flow (10).

Yet, some species may evolve prezygotic isolating barriers and still experience substantial gene flow. This is particularly true in species whose gametes have a dispersal phase distinct from the adults, such as plants and broadcast spawners (11). In this case, mechanisms of prezygotic isolation that act on adult individuals may be insufficient to prevent gene flow by gametes. Such systems are ideal for testing the potential for divergent natural selection to maintain species boundaries because they exhibit high rates of hybridization (potential gene flow) but low rates of introgression (realized gene flow) (12, 13).

The radiation of desert shrubs in the genus *Encelia* (Asteraceae) is a powerful system for investigating ecological mechanisms of species maintenance. The 18 recognized *Encelia* species and subspecies are morphologically and physiologically diverse and occupy a variety of specialized edaphic and climatic niches (14–16). Species often occur in close geographic proximity to each other, separated only by edaphic transitions. Further, the same pollinators are frequently observed moving between sympatric and parapatric species, and all continental species are obligate outcrossers (16, 17). Therefore, the potential for gene flow between species in *Encelia* is high. Indeed, distinct hybrid zones are found between eight unique species pairs, and they are almost always narrow and limited to ecotones or zones of disturbance (18). No evidence for intrinsic barriers to reproduction has yet been found in experimental crosses between different taxon pairs in the genus (16, 17). This pattern is thus indicative of extrinsic or ecological control over hybrid zone structure (1, 19).

**Fig. 1.**
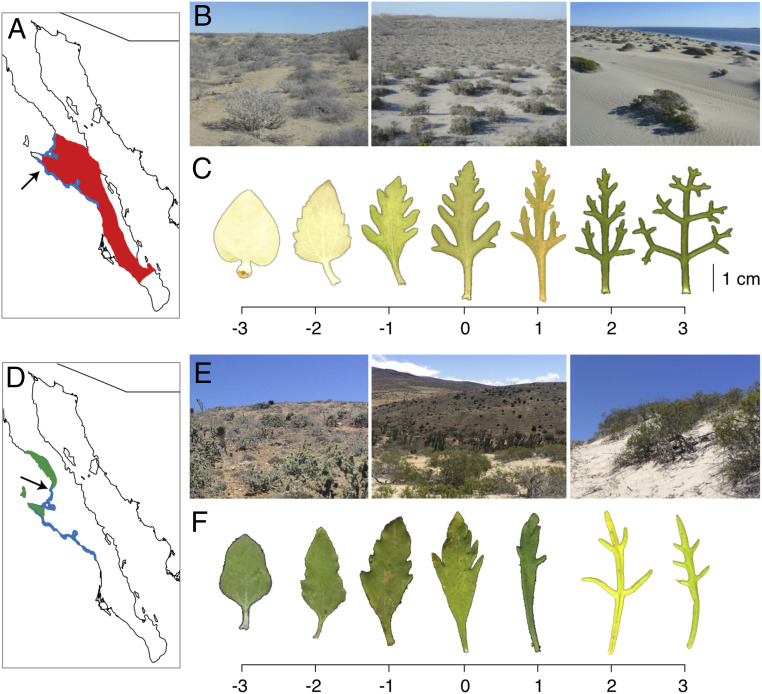
(*A*) Map of Baja California Sur showing the range of *E. palmeri* (red) and *E. ventorum* (blue), with the San Roque experimental site marked by the arrow. (*B*) Photographs of (*Left*) desert, (*Center*) ecotone, and (*Right*) dune habitats at San Roque. (*C*) Representative leaf phenotypes of (*Left*) *E. palmeri*, (*Right*) *E. ventorum*, and (*Center*) hybrids. Scale bar at the bottom is a multivariate hybrid index composed of the first axis of a principal components analysis of leaf shape and area. (*D*) The range of *E. asperifolia* (green) and *E. ventorum* (blue), with the Punta Lobos sampling site marked by the arrow. (*E*) Photographs of (*Left*) desert, (*Center*) ecotone, and (*Right*) dune habitats at Punta Lobos. (*F*) Representative leaf phenotypes of (*Left*) *E. asperifolia*, (*Right*) *E. ventorum*, and (*Center*) hybrids.

The hybrid zones between *Encelia palmeri*–*Encelia ventorum* (hereafter, *palmeri*–*ventorum*) and *Encelia asperifolia*–*E. ventorum* (hereafter, *asperifolia*–*ventorum*) illustrate these patterns particularly well. These species are endemic to the Baja California Peninsula of México where *E. ventorum* inhabits coastal sand dunes and *E. palmeri* and *E. asperifolia* occupy inland desert plains (Fig. 1). Across both pairs of hybridizing taxa, species exhibit morphological divergence that putatively reflects adaptation to their contrasting environments. Although species are strictly limited to their respective habitats, hybrid zones form at the linear ecotones between dune and desert habitats (*SI Appendix*, Fig. S1). For example, previous data from one *palmeri*–*ventorum* hybrid zone showed that hybrids are narrowly limited to the ecotone and that the interspecific hybridization rate for plants ∼200 m from the ecotone is 3.6% (16). These hybrids are vigorous and produce large quantities of viable propagules, yet parental taxa have maintained adjacent distributions in some locations for at least 125 y without fusing (16, 20). Based on these observations, we hypothesized that strong natural selection maintains adaptive divergence despite high rates of hybridization. We tested this hypothesis by asking the following three questions. 1) What is the magnitude and direction of hybridization and introgression between taxa in each of the two hybrid zones? Then, for the *palmeri*–*ventorum* hybrid zone, we further ask the following. 2) What is the magnitude and direction of phenotypic selection in dune, desert, and ecotone habitats? 3) What are the ecological mechanisms generating selection on hybrid and parental phenotypes?

## Results and Discussion

### Patterns of Gene Flow.

#### Hybridization or potential gene flow.

**Fig. 2.**
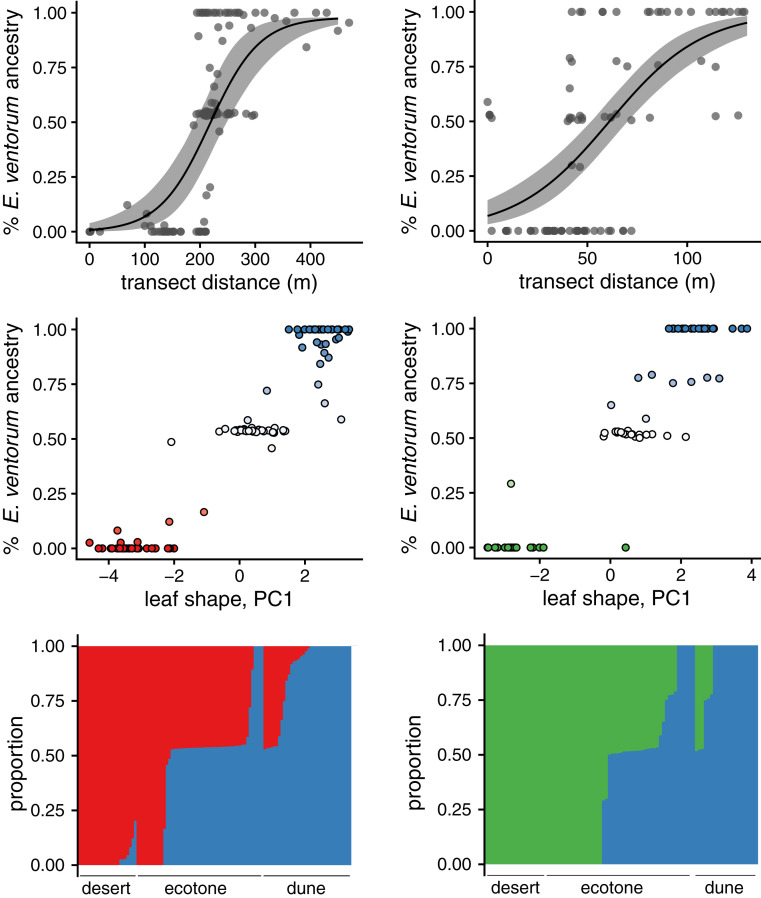
Patterns of hybridization and introgression at two hybrid zones in *Encelia*. (*Left*) *E. palmeri*–*E. ventorum* (*n* = 112 individuals across 309,000 single-nucleotide polymorphism [SNPs]); (*Right*) *E. asperifolia*–*E. ventorum* (*n* = 91 across 573,000 SNPs). (*Top*) Inference of cline width based on genomic ancestry estimates, (*Middle*) the distribution of individuals in phenotypic and genetic space, and (*Bottom*) inference of genomic ancestry for individuals in the hybrid zone based on variant data from ddRAD loci. Ancestry results are shown ordered by habitat and then ancestry proportion; red is *E. palmeri*, green is *E. asperifolia*, and blue is *E. ventorum*. Across both hybrid zones, we see narrow clines given effective dispersal of these species, limited introgression beyond the hybrid zone, and evidence that most hybrids are F1s. Together, these results suggest extremely strong selection is structuring these hybrid zones and thus, also maintaining species boundaries between these hybridizing pairs.

To estimate the potential for gene flow between *palmeri*–*ventorum* and *asperifolia*–*ventorum*, we genotyped and phenotyped 112 and 91 adult individuals, respectively, across parental habitats and the ecotone transition. Across both taxon comparisons, genetic and morphological indices of admixture were highly correlated (*r* = 0.95, *P* = 1e-58 and *r* = 0.94, *P* = 6e-30 for *palmeri*–*ventorum* and *asperifolia*–*ventorum*, respectively) (Fig. 2 and *SI Appendix*, Fig. S2). Using a maximum likelihood approach that categorizes hybrids based on genetic data (21), we found that 72% (*n* = 54 for *palmeri*–*ventorum*) and 50% (*n* = 52 for *asperifolia*–*ventorum*) of plants in the ecotones were hybrids (*SI Appendix*, Figs. S3 and S4). Of these hybrids, 72 and 71% were first-generation (F1s) in the *palmeri*–*ventorum* and *asperifolia*–*ventorum* hybrid zones, respectively.

In addition, for *palmeri*–*ventorum*, we estimated the rate of present-day hybridization. To do so, we sampled seeds from naturally occurring, phenotypically pure parental plants located within ∼200 m of either side of the ecotone, germinated these seeds in a greenhouse, and measured the percentage of offspring that were hybrids based on morphological indices. We found that 31.4% (*n* = 175) of *E. palmeri* progeny and 5.6% (*n* = 409) of *E. ventorum* progeny showed evidence of interspecific hybridization or backcrossing (*Estimates of Current Hybridization*). This asymmetry in hybridization rate might partially reflect the different flowering phenologies of the two species. Like many desert species (22), both species flower primarily in response to rainfall. However, *E. ventorum* has an extended flowering period because of the greater water availability in the dunes (16). Thus, *E. ventorum* is protected from interspecific pollen flow for part of its flowering period, while the flowering period of *E. palmeri* completely overlaps with that of *E. ventorum*, leading to greater potential for interspecific gene flow. This asymmetry acts as a form of phenological isolation, which along with other potential sources of prezygotic isolation such as geographic or habitat isolation, likely reduces hybridization rates. However, despite this prezygotic isolation, hybrids still dominate the hybrid zone, and many seeds outside of the hybrid zone show evidence for admixture, suggesting potential gene flow into and across the hybrid zone is high.

#### Introgression or realized gene flow.

**Fig. 3.**
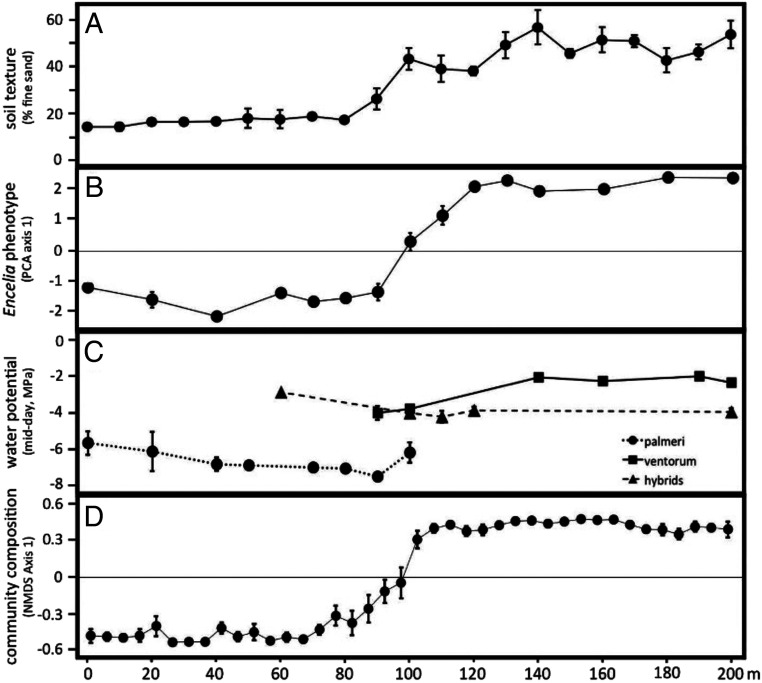
Transects centered on and perpendicular to the ecotone for the *E. palmeri*–*E. ventorum* hybrid zone reveal concordant clines in (*A*) soil texture, (*B*) *Encelia* leaf morphology, (*C*) *Encelia* midday stem water potential, and (*D*) species composition of the rest of the perennial plant assemblage. The transect begins in the desert (*E. palmeri* habitat) and ends in the dune (*E. ventorum* habitat). Measurements were performed along seven replicate 200-m transects. PCA: principal component analysis; NMDS: non-metric multidimensional scaling. Error bars are ±1 SE.

In contrast to potential gene flow, we inferred low levels of introgression beyond the hybrid zones between *palmeri*–*ventorum* and *asperifolia*–*ventorum*. Using 309K and 573K genetic variants from the *palmeri*–*ventorum* and *asperifolia*–*ventorum* hybrid zones, respectively, we characterized the extent of introgression in both hybrid zones. Across both hybrid zones, we found similar patterns: narrow clines coincident with habitat transitions, limited evidence for introgression beyond the ecotone, and an abundance of F1 hybrids (Fig. 2 and *SI Appendix*, Figs. S3 and S4). Cline widths were estimated as 118 and 93 m for *palmeri*–*ventorum* and *asperifolia*–*ventorum*, respectively. Approximately 200 m from the hybrid zone, 5 to 30% of seeds show evidence of hybridity, yet we see no adult hybrid individuals at this distance. Although F1 hybrids are formed often, hybrids rarely act as a bridge for gene flow, leading to only limited and spatially restricted introgression between the parental taxa. Indeed, almost all hybrids we identified form in the ecotone or at the ecotone edge but rarely in the habitat of the parental species (*SI Appendix*, Fig. S4). This discrepancy between potential and realized gene flow suggests divergent natural selection prevents introgression outside of the ecotone, thus maintaining species boundaries despite ongoing hybridization. The *palmeri*–*ventorum* hybrid zone coincides with a steep gradient in soil type and with a near-complete turnover of the composition of the rest of the perennial plant assemblage (Fig. 3). Given the overlap between the hybrid zone and the habitat transition, we hypothesized that habitat-mediated selection is likely structuring this hybrid zone.

### Patterns of Natural Selection.

**Fig. 4.**
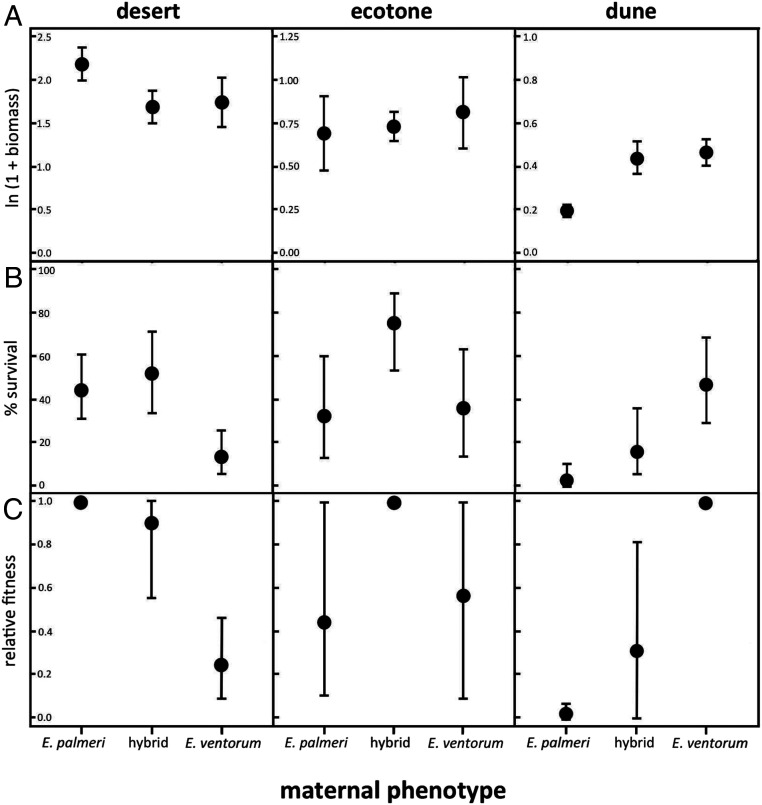
Results of the reciprocal transplant field experiment illustrating (*A*) growth measured as total ln-transformed biomass, (*B*) percentage survival to the end of the experiment, and (*C*) composite relative fitness obtained by multiplying growth and proportional survival and setting the most fit phenotype in each habitat equal to one. Error bars for biomass are ±1 SE, error bars for survival are binomial 90% CIs, and error bars for relative fitness are bootstrapped 95th percentile CIs truncated to between zero and one (*Reciprocal Transplant*).

To test the hypothesis that strong habitat-mediated selection is preventing introgression beyond the ecotone, we conducted a reciprocal transplant field experiment in one of the two hybrid zones: the *palmeri*–*ventorum* hybrid zone at the San Roque experimental site. This field-based experiment allowed us to measure the direction and magnitude of natural selection acting in dune, ecotone, and desert habitats (*Reciprocal Transplant* and *SI Appendix*, Fig. S1). Parental taxa and hybrids were grown from seed, collected, and transplanted into dune, ecotone, and desert habitats; they were watered for 2 mo and then allowed to grow naturally for 5 mo more. Aboveground and belowground biomass and survival were measured at the end of the experiment. This experiment revealed extremely strong divergent natural selection (Fig. 4), which is on par with some of the strongest natural selection measured between naturally hybridizing taxa (10, 23–25). Significant phenotype by habitat interactions were found for growth, survival, and composite fitness (*SI Appendix*, Table S1), with selection coefficients against parental migrants ranging from *s* = 0.755 for *E. ventorum* in the desert to *s* = 0.983 for *E. palmeri* in the dunes.

Hybrids were selected against in both parental habitats, with selection coefficients of *s* = 0.702 in the dune habitat and *s* = 0.097 in the desert habitat. In contrast, in the ecotone habitat, hybrids outperformed the parental species (*E. palmeri*; *s* = 0.582 and *E. ventorum*; *s* = 0.460), although this difference was not statistically significant. Theory predicts that hybridization can lead to both negative genetic interactions (e.g., heterozygote disadvantage or negative epistasis) or heterosis (26, 27). In *Encelia*, hybrid fitness seems to be contingent on the environment. Hybrids’ growth and survival are greater than the parent species in the ecotone (hybrid vigor) and comparable in the parental habitats. This pattern confirms experimental evidence that suggests few intrinsic incompatibilities in hybrids (16). In parental habitats, however, hybrids show reduced fitness relative to parental species, restricting the number of F1 hybrids and later-generation recombinants found in these habitats (*SI Appendix*, Figs. S3 and S4). This variance in hybrid fitness across habitats is consistent with other studies of natural plant hybridization [e.g., *Iris*, *Artemisia*, and *Helianthus* (28, 29)].

Seventy percent of hybrids in the ecotones are F1s (*SI Appendix*, Figs. S3 and S4); most hybrid zones in other plant species are comprised of more backcrosses than evidenced here (19, 30). F1-dominated hybrid zones can emerge if secondary contact between species occurred in the last generation, if relative population sizes of parental species and hybrid individuals make backcross matings rare, and/or if parental taxa and other hybrid classes are unable to thrive in the ecotone (31, 32). This hybrid zone is at least ∼125 y old (16, 20), or about five generations old, so recency of contact cannot explain the structure of this hybrid zone. Instead, the dominance of F1s must result from a combination of demographic factors and/or selection against later-generation hybrids. Selection on later-generation hybrids is likely complex: compared with F1 hybrids, they are assumed to experience less heterosis, to harbor either more or fewer genetic incompatibilities depending on dominance of epistatic loci, and to see breakdowns of coadapted gene complexes that might allow survival in the unique environment of the ecotone (31, 33–35). The set of hybrid plants used in our transplant experiment represented a range of hybrid ancestry (*SI Appendix*, Fig. S5), preventing us from measuring how fitness varies across hybrid generation. However, our results suggest, as has been shown in a number of other hybrid zones (31, 32, 36, 37), that F1s can have high fitness—to the exclusion of other genotypes—in ecotones.

Many reciprocal transplant experiments are conducted in herbaceous, annual plants (24, 38), in which the span of a typical experiment captures most of the life cycle of an individual. In contrast, *Encelia* are shrubs projected to live ∼10 to 25 y (39). Therefore, we could only census the first 5 mo of life of these long-lived plants. These first 5 mo, however, capture the most challenging time for these organisms because during this critical window of time, seedlings must recruit and establish in the face of extreme heat and dry soil in the desert and shifting sands on the dune. In fact, survival rates were <10% for both *E. palmeri* and *E. ventorum* in their nonnative habitats (Fig. 4*B*). Thus, selection acting early in the life cycle likely has a disproportionate effect on total lifetime fitness. However, given that these species reproduce continuously, later-stage selection could mediate the fitness differences we find here. Long-running reciprocal transplant experiments have shown that fitness differences between species can be both heightened and attenuated through time (11, 28, 40).

The results from the transplant experiment suggested that selection acts throughout the critical first 5 mo of the life cycle of these plants, against both parental migrants and hybrids, helping prevent introgression beyond the ecotone. If parental seeds disperse into the nonnative habitat, they are unlikely to survive (Fig. 4), leading to parental species largely being restricted to their native habitat and reducing the frequency of hybridization (*SI Appendix*, Figs. S3 and S4) (4, 6, 13, 41). The parental species thus exhibit prezygotic isolation in the form of habitat isolation (13), which is compounded by the effects of geographic isolation and the asymmetry in flowering times. However, this prezygotic isolation is insufficient to stop gene flow at the hybrid zone because pollen can still disperse across habitat boundaries. This leads to high rates of cross-pollination such that 5 to 30% of seeds in each parental habitat are admixed, as far as 200 m from the edge of the hybrid zone. However, these admixed individuals are selected against relative to native parental phenotypes (Fig. 4), resulting in postzygotic extrinsic selection that helps limit introgression beyond the hybrid zone (Fig. 2). The delay between gene flow via pollen and the onset of reproduction creates a long prereproductive period in which natural selection can act, further enhancing habitat isolation (42). Future studies might identify other potential sources of isolation—including phenological isolation between hybrids and their parents or hybrid breakdown in backcrosses—that further act to restrict introgression.

Thus, our results suggest that the habitat selects against both parental migrants and hybrids similarly (6, 43), and this habitat selection helps maintain species boundaries in these hybridizing species. Given estimated hybridization rates, these species should fuse as alleles introgress deeper into parental populations across generations. In contrast, sharp phenotypic and genetic boundaries are maintained over the scale of meters, as has been seen in other species pairs that exhibit strong local adaptation to narrow environmental gradients (11, 44).

### Mechanisms of Natural Selection.

The desert and dune environments differ in water availability, wind strength, soil salinity, and herbivory pressure, which we hypothesized creates selection gradients between the two habitats and contributes to the patterns of selection measured in our reciprocal transplant experiment.

#### Water availability.

The desert habitat is drier, hotter, and less humid than the dune habitat (*SI Appendix*, Figs. S6 and S7). The coarser texture of dune sand results in more rapid water infiltration, allowing precipitation from fog, dew, and small rain events to percolate deeper than in adjacent desert soils (*SI Appendix*, Fig. S6) (45). After water infiltrates, it is strongly insulated from evaporation, creating a persistent lens of water suspended within the dune that is a common feature of both coastal and inland dune systems (46, 47).

**Fig. 5.**
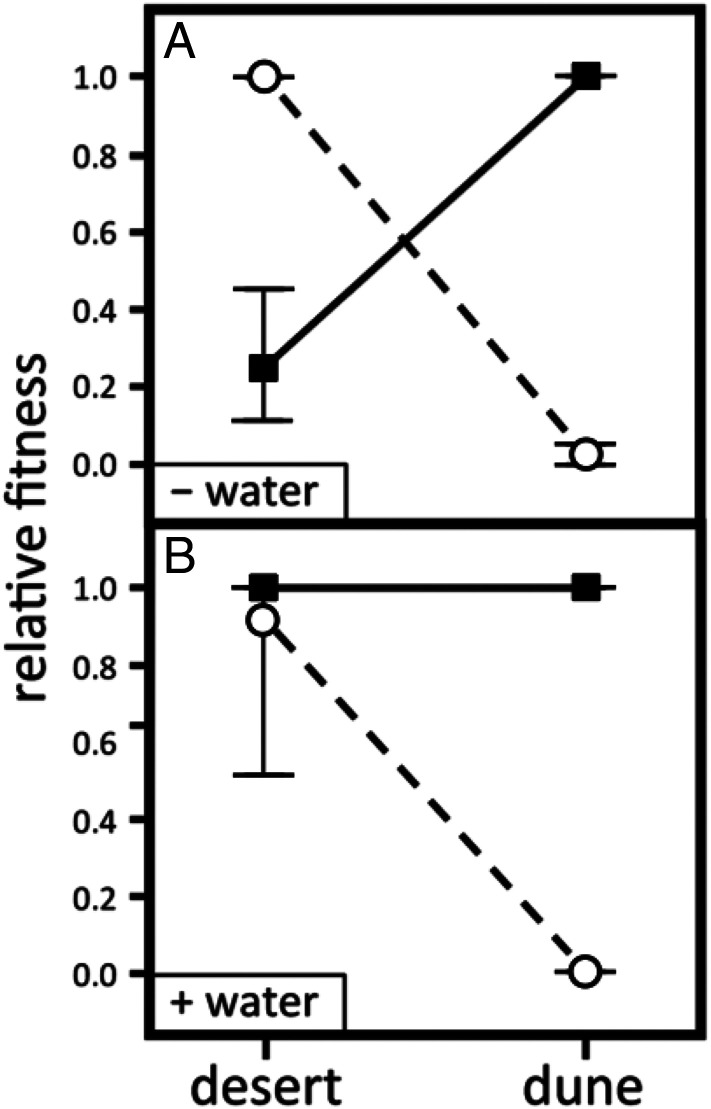
Relative fitness of *E. palmeri* (circles) and *E. ventorum* (squares) in dune and desert habitats (*A*) without supplemental water and (*B*) with supplemental water. Relative fitness is calculated proportionally, with the most fit taxon in each habitat set to one. Error bars are bootstrapped 95th percentile CIs truncated to between zero and one.

We experimentally tested the importance of this gradient in water availability by adding water to a subset of the plants in the reciprocal transplant experiment (*Methods*). Increasing water availability equalized fitness differences in the desert habitat, eliminating divergent natural selection (Fig. 5). By contrast, adding water had no effect on growth or survival of either species in the dune habitat (*SI Appendix*, Table S1). These results show that the gradient in water availability between habitats is necessary but not sufficient to cause divergent natural selection.

Hydraulic traits of *E. palmeri*, *E. ventorum*, and hybrids differed in ways that are consistent with published studies on plant adaptation to drought (*SI Appendix*, *Hydraulic Physiology*). Xylem vessel diameter, stem hydraulic conductance, leaf hydraulic capacitance, and midday stem water potential were all predicted to be lower in *E. palmeri* from xeric desert habitats than *E. ventorum* from mesic dune habitats (48, 49). Xylem vessel diameter varied in the predicted directions, with vessel diameter smallest in *E. palmeri*, largest in *E. ventorum*, and intermediate in hybrids (*SI Appendix*, Fig. S8 and Table S2) (one-way ANOVA: *P* = 0.013, *F* = 4.36, *df* = 2). Leaf hydraulic capacitance also varied in the predicted directions, with capacitance lowest in *E. palmeri*, greatest in *E. ventorum*, and intermediate in hybrids (*SI Appendix*, Fig. S9). Midday stem water potential at the center of the hybrid zone also varied in the predicted directions, with *E. palmeri* exhibiting more negative water potentials than hybrids or *E. ventorum* (Fig. 3*C*) (one-way ANOVA: *P* < 0.001, *F* = 23.4, *df* = 27). Stem hydraulic conductance was the only trait that did not vary in the predicted direction, with *E. palmeri* exhibiting greater conductance per unit leaf area than *E. ventorum* (*SI Appendix*, Table S2) (two-sample *t* test: *P* = 0.046, *F* = 2.61, *df* = 5). However, desert shrubs have the highest leaf-specific shoot hydraulic conductance of all woody plants, likely because their window of physiologically amenable conditions is very narrow (50) (*SI Appendix*, Fig. S7).

#### Wind.

The dunes inhabited by *E. ventorum* are created by strong onshore winds that transport beach sand inland (51, 52); the specific epithet *ventorum* translates as “blowing winds.” These winds create stable ecotones between dune and desert habitats defined by the tangent of the direction of the wind to the curvature of the beach (*SI Appendix*, Fig. S1) (53, 54). A variety of biomechanical stresses is generated by wind including abrasion of the leaf cuticle and structural failure from flapping (55). Additionally, wind results in burial and excavation of whole plants as dunes shift vertically and laterally (56). We tested the importance of wind as a component of natural selection by scoring mortality due to burial in the reciprocal transplant experiment (*Reciprocal Transplant*). A plant was scored as killed by burial if it was completely covered with sediment, exhibited dry or necrotic tissues when excavated, and had been healthy at previous time points (*SI Appendix*, Fig. S10). Analysis of mortality due to burial revealed a significant habitat by phenotype interaction (*SI Appendix*, Table S1). Mortality from burial was highest in the dunes (22.2%), less in the ecotone (7.0%), and not observed in the desert habitat (0%). Across all three habitats, mortality from burial only affected *E. palmeri* (17.5%) and hybrids (9.0%), with no individuals of *E. ventorum* dying from burial during the course of the experiment (*SI Appendix*, Fig. S11*B*).

Several morphological traits unique to *E. ventorum* may function as adaptations to wind and burial. First, *E. ventorum* exhibits heavily dissected, glabrous leaves (Fig. 1), traits that in other species reduce aerodynamic drag and dampen the flapping instability that can cause structural failure (55). Second, *E. ventorum* exhibits pronounced juvenile apical dominance relative to *E. palmeri*, resulting in a narrow, conical canopy architecture (*SI Appendix*, Fig. S12) that is known to function as an adaptation to avoid burial (56, 57). Finally, unlike other species in the genus, *E. ventorum* exhibits adventitious rooting, which is known to function as an adaptation to track shifting sand dunes (56).

#### Salinity.

Salt spray is a major stressor in many coastal habitats (58). To test for a gradient in salt stress, we first quantified variation in the degree of leaf succulence, a known response to salinity that functions to dilute the internal salt concentration of leaves (59). Using leaf thickness as a proxy for succulence, we measured the thickness of leaves along a transect between dune and desert habitats and also around the circumference of a large *E. ventorum* individual growing on the exposed foredune (*SI Appendix*, *Hydraulic Physiology*). Consistent with our predictions, leaf thickness was two to three times greater in the foredune habitat than near the desert (*SI Appendix*, Fig. S13*B*) and was also two to three times greater on the windward, ocean-exposed side of the canopy compared with the leeward side (*SI Appendix*, Fig. S13*C*).

Further, we might expect plants closer to the ocean to show greater evidence of salt stress than plants farther from the ocean. Accordingly, we measured leaf osmotic potential of the storage parenchyma cells in *E. ventorum* leaves along a transect from desert to dune. We hypothesized that leaf osmotic potential would become more negative with increasing proximity to the ocean because salt is passively taken up by the roots and because salt deposition on the leaves is a function of exposure to onshore winds (59, 60). Leaf osmotic potential was measured by excising and macerating internal nonphotosynthetic parenchymatous tissue under a saturated atmosphere and measuring the water potential of the macerated tissue using thermocouple psychrometers (*SI Appendix*, *Hydraulic Physiology*). This method eliminates remaining xylem tension and turgor pressure, so that the residual water potential is equal to the osmotic potential. Consistent with our hypothesis, leaf osmotic potential decreased to more negative values from the desert to the foredune, indicating that salt stress increases with proximity toward the ocean (*SI Appendix*, Fig. S13*A*) (linear regression: *P* < 0.01, *R*^2^ = 0.80, *n* = 8).

**Fig. 6.**
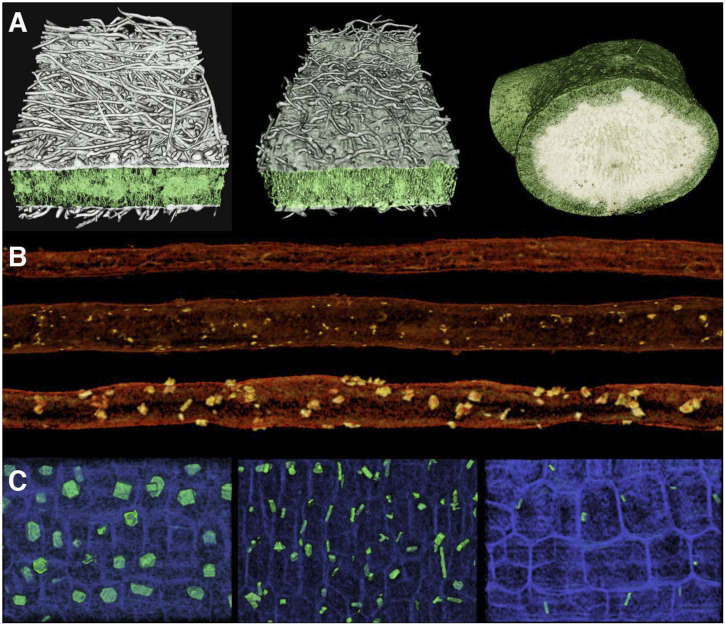
(*A*) The 3D oblique-transverse images of leaf laminas of (*Left*) *E. palmeri*, (*Center*) a hybrid, and (*Right*) *E. ventorum* obtained using microCT imaging. In *E. ventorum*, the lightly colored cells in the center of the leaf are storage parenchyma holding salt. Exposed transverse surfaces are 820-, 820-, and 2,000-µm wide, respectively. (*B*) Representative 3D microCT images of resin ducts of (*Top*) *E. palmeri*, (*Middle*) a hybrid, and (*Bottom*) *E. ventorum*, showing differences in size and abundance of associated crystals. Resin ducts shown are all 1-mm long. (*C*) Representative microCT images of stem pith cells (blue) of (*Left*) *E. palmeri*, (*Center*) a hybrid, and (*Right*) *E. ventorum*, highlighting differences in the size, shape, and abundance of pith crystals (green). All images are 300 by 200 µm.

Additional evidence for the prevalence of salt stress comes from three-dimensional (3D) X-ray microcomputed tomography (microCT) reconstructions of the stems of parental taxa and hybrids, which revealed the presence of irregular crystals inside and around resin ducts (Fig. 6*B*). These crystals were abundant in *E. ventorum*, rare in *E. palmeri*, and present in intermediate amounts in hybrids (*SI Appendix*, Table S2) (one-way ANOVA: *P* < 0.001, *F* = 18.09, *df* = 2). Repeated microCT imaging of the stems as they desiccated revealed that these ducts are normally hydrated and that the crystals form as the stem desiccates. Since active salt sequestration in stems is a known adaptation to salinity in other taxa (59, 60), we hypothesized that these crystals were condensed ocean-derived salts. To test this hypothesis, we measured the X-ray mass attenuation coefficients (MACs) of crystals from parental taxa and hybrids and compared them with the MACs of a number of candidate compounds (*SI Appendix*, Table S3). The crystals associated with resin ducts had average MACs ranging from 7.16 to 7.51, placing them within the range of the MACs of the solid forms of the major salt constituents of seawater (NaCl = 6.26, MgCl_2_ = 7.95, KCl = 10.12). In comparison, several chromenes and benzofurans, known constituents of the resins in *Encelia* (61), have MACs less than 0.2. Other compounds were also poor matches, including silicon dioxide (SiO_2_ = 2.73) and calcium oxalate (CaC_2_O_4_ = 5.17), two secondary compounds frequently found in crystalline form in plants (62).

#### Herbivory.

A variety of herbivores was observed consuming *Encelia* plants at the San Roque experimental site. *E. palmeri* and *E. ventorum* share some of the same pollinators (16), so some insects move between the two habitats. However, given the near-complete turnover of the plant community across the two habitats (Fig. 3*D*), the insect community composition likely experiences turnover too. To determine whether herbivory contributed to divergent selective pressures, we first analyzed sources of mortality in the reciprocal transplant experiment (*SI Appendix*, Fig. S11*A*). Plants were diagnosed as having been killed by herbivory if they were completely defoliated, if tracks or other signs of herbivore activity were found nearby, and if the plant was otherwise healthy at previous time points (*Reciprocal Transplant*). Analysis of patterns of herbivory and mortality indicated a significant habitat by phenotype interaction (*SI Appendix*, Table S1). In the desert habitat, *E. ventorum* was the most heavily consumed (23%), followed by hybrids (8%) and *E. palmeri* (2%). In the dune habitat, *E. palmeri* and *E. ventorum* both sustained 14% mortality from herbivory, while no hybrids were killed by herbivory.

Additionally, due to the higher resource availability in the dune habitat and the higher growth rate of *E. ventorum*, we predicted that *E. ventorum* would exhibit lower investment in secondary compounds and structures than *E. palmeri* due to a trade-off between investment in growth and herbivore defense (63). One conspicuous difference between *E. ventorum* and *E. palmeri* is the presence of a dense pubescence of trichomes on the leaves of *E. palmeri* and hybrids, which are known to function as a mechanical defense against herbivory (64). Although trichomes may have other functions, including reflecting solar radiation and increasing boundary-layer thickness (48), the density of trichomes varied in the directions predicted based on patterns of herbivory (Fig. 6*A*) (*E. palmeri* = 447.6 ± 9.1 mm^−2^, hybrids = 84.6 ± 13.0, *E. ventorum* = 0.17 ± 0.17; one-way ANOVA: *P* < 0.001, *F* = 224.7, *df* = 2).

*Encelia* species are also known for their diversity of secondary compounds, many of which are known defenses against herbivory (61, 65). In *E. palmeri*, microCT analysis revealed that the pith cells of the stems contained a large number of conspicuous crystals that differed in shape and location from those previously identified in the resin ducts (Fig. 6*C*). These crystals were most abundant in *E. palmeri*, less frequent and smaller in hybrids, and very infrequent and smallest in *E. ventorum* (*SI Appendix*, Table S2) (one-way ANOVA on abundance: *P* < 0.001, *F* = 217.1, *df* = 2). The MACs of these crystals (*E. palmeri* = 5.17 ± 0.15, hybrids = 5.32 ± 0.11, *E. ventorum* = 5.19 ± 0.11) closely matched the MAC of calcium oxalate (CaC_2_O_4_ = 5.17), a secondary compound known to act as a mechanical and chemical deterrent to herbivory in a large number of plant taxa (62). Together, these results indicate that herbivory likely contributes to divergent selective pressures but that further study is needed to disentangle the multiple sources of selection acting on secondary compounds and structures.

#### Multiple sources of selection.

Taken together, our findings show how strong ecological trade-offs can be created through multiple agents of selection; collinear gradients of water, burial, salinity, and herbivory all contribute to divergent natural selection. Based on mortality patterns (*SI Appendix*, Table S1), each factor either operated strongly in one habitat but was neutral in the other or operated in the same direction in both habitats but stronger in one. These data suggest that while the net effect of all sources of selection was divergent, no single factor alone exhibited divergent selective pressures. The net effect of these selection gradients seems to have resulted in total stronger selection in the dunes than the deserts. Based on the transplant experiment, we estimate that *E. palmeri* experienced stronger selection in the dunes (*s* = 0.983) than *E. ventorum* did in the deserts (*s* = 0.755). Hybrids show a similar pattern (*s* = 0.702 in the dunes vs. *s* = 0.097 in the deserts). This might reflect that the stressors of the dunes (e.g., high salinity and wind speed) create absolutely stronger gradients than those of the deserts (e.g., water availability and herbivory). Properly disentangling the relative direction and magnitude of these different selective agents will require controlled isolation of each factor and then testing its effects across species in a common environment and across multiple growing seasons.

Although we have yet to identify the ecological mechanisms of selection acting in the *asperifolia*–*ventorum* hybrid zone, the similarities between the two hybrid zones suggest similar processes are structuring this hybrid zone as well (Fig. 2). Thus, *Encelia* builds on other studies that show that ecologically divergent evolutionary lineages maintained by selection may be more common than is appreciated, especially if adaptation involves adjustments in traits that are hard to measure or not readily visible and involves multiple ecological gradients simultaneously (4, 31, 32, 66, 67).

## Conclusion

Using a combination of fine-scale genetic and phenotypic characterizations of hybrid zones, manipulative field experiments, 3D anatomical visualizations, and ecophysiological measurements, we have shown how ecologically based divergent natural selection maintains phenotypic and genetic divergence between codistributed species despite widespread hybridization. Our study further provides explicit ecological mechanisms for four different selective agents including water availability, salinity, burial, and herbivory, underscoring that adaptation to different habitats likely involves complex interactions between multiple agents of selection acting on whole phenotypes. Natural hybrid zones associated with habitat transitions can be found between eight species pairs in *Encelia* (18), yet no cases of phenotypic fusion or species collapse are known. Our study thus provides a mechanistic basis for the rapid generation of diversity in *Encelia* as it radiated throughout the deserts of North and South America and showcases the potential power of natural selection in generating and maintaining biodiversity.

## Methods

### Overview.

We first used fine-scale phenotypic and genotypic sampling to determine how hybridization patterns change across narrow ecotones between two pairs of desert plant species, *E. palmeri*–*E. ventorum* and *E. asperifolia*–*E. ventorum*. Concordant patterns across both hybrid zones justified focus on one pair: *palmeri*–*ventorum*. For this pair, we used a reciprocal transplant experiment to test the hypothesis that strong natural selection was structuring the hybrid zone. After supporting our hypothesis, we conducted fine-scale analyses of the anatomy and physiology of *palmeri*–*ventorum* to determine possible mechanisms of selection. Full details on our methods are available in *SI Appendix*.

### Site Characteristics.

All sampling, field measurements, and experiments for *palmeri*–*ventorum* were carried out at San Roque, a settlement on the Pacific Coast of Baja California Sur, México (Fig. 1). All sampling for *asperifolia*–*ventorum* was carried out on Punta Lobos, a site on the Pacific Coast of Baja California Sur, México (Fig. 1). Both sites are characteristic of the sand dune–desert ecotones found throughout the Baja California Peninsula. Afternoon winds across the peninsula are consistent and predictable (51, 52, 68) (*SI Appendix*, Fig. S14), which creates stable, linear ecotones between dune and desert habitats with dunes moving parallel to the ecotone (*SI Appendix*, Fig. S1). Habitat type can be identified by eye based on differences in soil color and texture that arise due to differential sand content between the desert and the dune (Fig. 1 *B* and *E*); desert soil comprises ∼15% fine sand, whereas dune soil comprises ∼55% fine sand (Fig. 3). The ecotone is defined as the narrow 20-m linear region at the interface between these habitats (Fig. 3*A*).

### Hybrid Zone Analysis.

To analyze patterns of hybridization and introgression at the *palmeri*–*ventorum* and *asperifolia*–*ventorum* hybrid zones, we analyzed phenotypic and genetic data from adult plants across transects in each hybrid zone (*SI Appendix*, Fig. S15). We sampled adult individuals along a linear transect through the hybrid zone, recording latitude, longitude, and habitat type for each individual. In the ecotone, we sampled perpendicular to the transect due to the narrowness of the transition. In total, we sampled 112 and 91 individuals for the *palmeri*–*ventorum* and *asperifolia*–*ventorum* hybrid zones, respectively.

To characterize genetic patterns at the hybrid zones, we collected double-digest restriction-aided (ddRAD) data from each individual. Per hybrid zone, we assembled loci and called variants across all individuals using Velvet (69), vsearch v2.4.3 (70), bwa v0.7.17 (71), and ANGSD v0.923 (72). To determine patterns of admixture and introgression across individuals, we used NGSadmix v32 to calculate ancestry proportions for each individual (73) and snapclust to classify individuals as either parental, F1 hybrids, or backcrosses (21). We used HZAR to estimate genetic clines based on ancestry proportions (74). To accommodate our sampling design, we binned individuals into 10-m bands running perpendicular to the transect (*SI Appendix*, Fig. S15), calculating average genetic ancestry for each band (our “population”).

To collect and analyze phenotypic data from the hybrid zones, we measured leaf area and shape by photographing leaves with a scale bar using a digital camera and then analyzing the images in ImageJ (US NIH; https://imagej.nih.gov/ij/). After conducting a scaled and centered principal component analysis on all leaves from a given hybrid zone, we averaged principal component (PC) scores per individual across all leaves measured for that individual. PC1 captured 52% of the variation in the *asperifolia*–*ventorum* hybrid zone and 45% of the variation in the *palmeri*–*ventorum* hybrid zone. In both hybrid zones, PC1 largely reflected leaf shape (Fig. 1 *C* and *F*).

### Estimates of Current Hybridization.

To obtain current field-based estimates of hybridization rates in *E. palmeri* and *E. ventorum*, we collected fruits (cypselae) from phenotypically pure individuals growing near the hybrid zone and assayed their progeny for traits indicative of interspecific hybridization. In 2016, we collected cypselae from several hundred *E. palmeri* and *E. ventorum* mothers and germinated them in indoor flats filled with potting soil. After germination, any seedling that visually scored between −1 and 1 on the hybrid index (Fig. 1*C*) was recorded as hybrid.

### Environmental Characterization of the *Palmeri*–*Ventorum* Hybrid Zone.

We characterized the abiotic and biotic conditions across the dune–desert ecotone in *E. palmeri*–*E. ventorum*. To measure microclimates, we used paired weather stations and soil moisture arrays to measure relative humidity, temperature, wind speed, leaf wetness, and soil moisture (*SI Appendix*, Figs. S6 and S7). In the dune, we additionally measured photosynthetic photon flux density and wind direction. To measure soil texture, we characterized the percentage of soil that was “fine sand” across 20 equidistant points across a 200-m-long transect (Fig. 3*A*). To characterize biotic conditions, we performed community surveys of all non-*Encelia* vascular perennial plant species across seven 10 × 200-m belt transects. We summarized the resulting community data matrix using the “vegdist” function in the “vegan” package and the “isoMDS” function in the “mass” package in R (Fig. 3*D*).

### Reciprocal Transplant.

We conducted a reciprocal transplant field experiment during 2010 to 2011 at the San Roque experimental site. In fall 2010, 276 parental and hybrid plants grown from seed were planted into dune, desert, and ecotone habitats. Because *Encelia* is slow to set seeds, we were unable to generate F1 seeds and instead, used seeds harvested from hybrid mothers. These seeds spanned a range of phenotypes (*SI Appendix*, Fig. S5), suggesting they include later-generation backcrosses. We measured growth, survival, and reproduction over the course of one growing season. In March 2011, all surviving plants were harvested and measured for plant size, leaf morphology, and (when possible) midday shoot water potential (*Hydraulic Physiology*).

Growth was measured as total above- plus belowground biomass at the end of the experiment, or the total biomass of the plant at the time of its death. Survival was calculated as the percentage of plants that were alive at the end of the experiment. Only one plant from the water addition experiment flowered during the course of the experiment, so reproductive output was not analyzed. Growth and survival were additionally combined into a composite fitness measure by multiplying a binary (0, 1) survival score with the ln(1 + *x*)-transformed biomass. This composite measure is a reasonable approximation of fitness, given that total plant size is an important predictor of seed production (14). Relative fitness was calculated by standardizing by the phenotype with the highest fitness (Fig. 4*C*). Negative selection coefficients were calculated as one minus the relative fitness (25).

To test for the effects of habitat and phenotype on fitness, we constructed a general linear model using a negative binomial variance and link function implemented with the “glm.nb” function in R on ln(1 + *x*)-transformed data.

### Water Addition.

We tested the hypothesis that water availability affected relative fitness of *E. palmeri* and *E. ventorum* by adding supplemental water to a subset of the plants in the reciprocal transplant field experiment. Five plants of *E. palmeri* and eight plants of *E. ventorum* were randomly chosen in each of dune and desert habitats to continue receiving water for an additional 3 mo after an initial 2-mo watering period. Following completion of the experiment, plants were harvested and assayed for biomass and leaf traits. To test the effects of the watering treatment on growth, we constructed a general linear model using a negative binomial link function using the glm.nb function in R, first specifying a model with a water × phenotype interaction and then specifying a model with water and phenotype independently added. Model fit was compared through a likelihood ratio (LR) test.

### Sources of Mortality.

We surveyed all experimental reciprocal transplant plants two or three times a week to record instances of mortality due to herbivory and from burial by windblown sand. Herbivory was scored if the entire plant was destroyed between visits, with evidence of herbivore activity nearby such as tracks or the herbivore itself, and no other proximate cause identified (e.g., bad weather). Mortality due to burial was scored if the entire plant was completely buried by sand and if all leaves were wilted and lacking turgor when excavated (*SI Appendix*, Fig. S10).

To analyze patterns of burial in the reciprocal transplant experiment, we performed backward stepwise log-linear analysis on three-way contingency tables. To test for a habitat by phenotype interaction, we constructed models as described for the “water addition” experiment and performed LR tests on the two best-fitting models (*SI Appendix*, Table S1). We repeated this analysis for patterns of herbivory.

### Hydraulic Physiology.

To characterize physiological differences between *E. palmeri* and *E. ventorum*, we measured five aspects of plant hydraulic physiology: leaf hydraulic capacitance, stem hydraulic conductance, midday shoot water potential, leaf succulence, and leaf osmotic potential. We focused on hydraulic physiology because the reciprocal transplant experiment suggested that water availability imposes a significant selection gradient on these taxa. Midday shoot water potentials presented in Fig. 3 were measured on mature, naturally occurring plants, whose locations were mapped using GPS and binned according to their distance from the hybrid zone. Water potentials were measured using a Scholander-type pressure chamber (PMS; Model 1000). All measurements were made between 13:00 and 15:00 h on sunny days. One healthy 10-cm-long shoot per plant was excised using a razor blade and immediately placed into the pressure chamber. Water potentials of distance-binned individuals were averaged with error bars representing one SE (Fig. 3*C*). Differences in water potential at the ecotone habitat were analyzed using one-way ANOVA.

### X-Ray MicroCT.

To characterize divergence in fine-scale leaf and stem anatomy, we obtained high-resolution 3D images of stem and leaf structure by performing hard X-ray microCT at the Advanced Light Source, Lawrence Berkeley National Laboratory (LBNL), Beamline 8.3.2 (75, 76). Stem samples of *E. palmeri*, *E ventorum*, and hybrids growing naturally in the field in San Roque in March 2015 were cut, kept in humid plastic bags, and transported to LBNL for microCT imaging within 4 d of sampling. Stem samples were allowed to slowly desiccate and imaged repeatedly. Leaf samples were collected from plants growing in cultivation at the Agricultural Operations Station, University of California, Riverside in June 2015 and transported to LBNL, keeping the leaves hydrated until imaging was performed within 36 h of sampling. All samples were placed on a rotating stage in the 24-keV synchrotron X-ray beam, and 1,025 two-dimensional projections were recorded as the sample rotated continuously from 0° to 180°. These raw tomographic projections were assembled using Octopus 8.7 (University of Ghent) and then analyzed in Avizo 8.1 software. The resolutions of the resulting images were 1.24 and 1.36 µm for stems and leaves, respectively.

For analysis of crystal structure in stems, we used 16-bit reconstructions. In Octopus 8.7, the MAC was calculated by manually drawing a line transect through 15 crystals of each morphological type in each taxon and averaging peak pixel values. Mean and SE for each crystal type in each phenotype were calculated for comparison with predicted MAC values for candidate crystal compounds obtained from an online database maintained by Argonne National Laboratory (https://11bm.xray.aps.anl.gov/absorb/absorb.php). We then surveyed the literature to build a list of candidate compounds and queried this database for predicted MAC values of each candidate compound. This list of candidate compounds included the most abundant salts in seawater; several secondary compounds previously found in *Encelia* resins, including benzofurans and benzopyrans; and other compounds commonly occurring in crystalized forms in plants (*SI Appendix*, Table S3).

## Supplementary Material

Supplementary File

## Data Availability

Code used in analysis is available in GitHub (https://github.com/singhal/encelia_transplant) (77). Raw ddRAD reads are available in Sequence Read Archive BioProject (accession no. PRJNA675657) (78). Individual genotypes and phenotypes from each hybrid zone are available in Figshare (https://doi.org/10.6084/m9.figshare.13211036.v1) (79).

## Change History

March 16, 2021: The license for this article has been updated.
